# Association between platelet levels and bleeding events in patients with acute-on-chronic liver failure treated with an artificial liver support system

**DOI:** 10.3389/fmed.2026.1718780

**Published:** 2026-01-29

**Authors:** Li Yang, Fang Chen, Lingyao Du, Yuanji Ma, Lang Bai, Hong Tang

**Affiliations:** 1 Center of Infectious Diseases, West China Hospital of Sichuan University, Chengdu, China; 2 Department of Infectious Diseases, People's Hospital of Jianyang City, Jianyang, China

**Keywords:** acute-on-chronic liver failure, artificial liver support system, bleeding event, risk factor, thrombocytopenia

## Abstract

**Background:**

Platelet reduction is associated with an increased risk of bleeding in patients with chronic liver disease. However, the association between platelet levels and bleeding events in patients with liver failure treated with an artificial liver support system (ALSS) remains unclear.

**Methods:**

This retrospective study included patients with acute-on-chronic liver failure (ACLF) who received ALSS treatment. Logistic and linear regression analyses were employed to assess the association between platelet levels and bleeding events during hospitalization and the relationship between sessions of ALSS treatment and platelet reduction rates.

**Results:**

We included 262 patients, of whom 56 (21.4%) experienced bleeding events during hospitalization. Baseline platelet levels in patients with bleeding events were significantly lower than those in patients without bleeding events (59.0 (39.0 ~ 89.3) × 10^9^/L vs. 88.5 (57.0 ~ 121.0) × 10^9^/L; *p* < 0.001). Baseline platelet levels were negatively associated with bleeding events (adjusted OR, 0.986; 95% CI, 0.976–0.996; *p* = 0.006), whereas platelet reduction rates and final platelet levels were not significantly associated with bleeding events (all *p* > 0.05). Compared to patients with baseline platelet grade 0, the risk of bleeding was significantly higher compared to those with baseline platelet grade 1 (adjusted OR: 3.21, 95% CI: 1.20 ~ 8.59, *p* = 0.002) and grade 2 (adjusted OR: 7.20, 95% CI: 2.28 ~ 21.43, *p* = 0.001), as well as in the combined group of grades 2 and 3 (adjusted OR: 8.43, 95% CI: 2.96 ~ 23.99, *p* < 0.001). The number of ALSS treatment sessions was not significantly associated with platelet reduction rates (*p* > 0.05).

**Conclusion:**

Patients with ACLF who underwent ALSS treatment with lower baseline platelet levels were at an increased risk of bleeding during hospitalization, whereas the platelet reduction rate was not independently associated with bleeding risk. These findings underscore the importance of baseline platelet count rather than platelet reduction for early risk stratification in patients with ACLF.

## Introduction

Acute-on-chronic liver failure (ACLF) is a clinical syndrome that occurs in patients with chronic liver disease and is triggered by various factors, leading to acute deterioration of liver function. This condition is characterized by worsening jaundice, coagulopathy, and other signs of acute decompensation (1). Patients may also experience hepatic encephalopathy, hepatorenal syndrome, and other extrahepatic organ failure (1). The short-term mortality rate of ACLF ranges from 50 to 90% (2). In the Asia-Pacific region, hepatitis B virus (HBV) infection is the predominant cause of ACLF (3). As an available therapeutic option, artificial liver support system (ALSS) treatment has been shown to effectively reduce serum total bilirubin, inflammatory factors, ammonia, and liver enzymes, thereby improving the clinical status and short-term prognosis (4–7).

Patients with ACLF may experience varying degrees of platelet (PLT) reduction due to factors such as splenic hyperfunction, sequestration of blood cells in the spleen, and reduced hepatic synthesis of PLT-producing factors (8). The degree of PLT reduction is correlated with the severity of chronic liver disease and cirrhosis (9–13). During ALSS treatment in patients with ACLF, the extracorporeal circuit and associated devices may activate PLTs and promote their adhesion, thereby contributing to a further decline in PLT levels. In general, when the PLT level falls below 50 × 10^9^/L, the risk of bleeding increases, red blood cell or platelet transfusions may be required, and mortality rates may also rise (14). Previous studies have shown that the occurrence of bleeding events in patients with chronic liver disease is associated with an increased risk of multi-organ dysfunction and a higher mortality rate (15). However, the association between PLT levels and bleeding events in patients with ACLF who undergo ALSS treatment remains unclear. This retrospective study assessed the association between PLT levels, changes, and bleeding events during hospitalization in patients with HBV-related ACLF (HBV-ACLF) who underwent ALSS treatment.

## Methods

### Study design

Patients who received ALSS treatment at the Center of Infectious Diseases, West China Hospital of Sichuan University, were consecutively recorded in a clinical database since January 2014. To investigate the association between PLT levels and bleeding events in patients with ACLF who underwent ALSS treatment, we conducted a retrospective study and enrolled these patients from December 2019 to December 2021. This study was approved by the Biomedical Research Ethics Committee of West China Hospital of Sichuan University (2023(1720)). All study procedures were performed following the ethical standards of the 1964 Declaration of Helsinki and its later amendments. The data were anonymized, and the requirement for informed consent was waived. The study was registered in ChiCTR2300077144.^1^

### Patients

**Figure 1:**
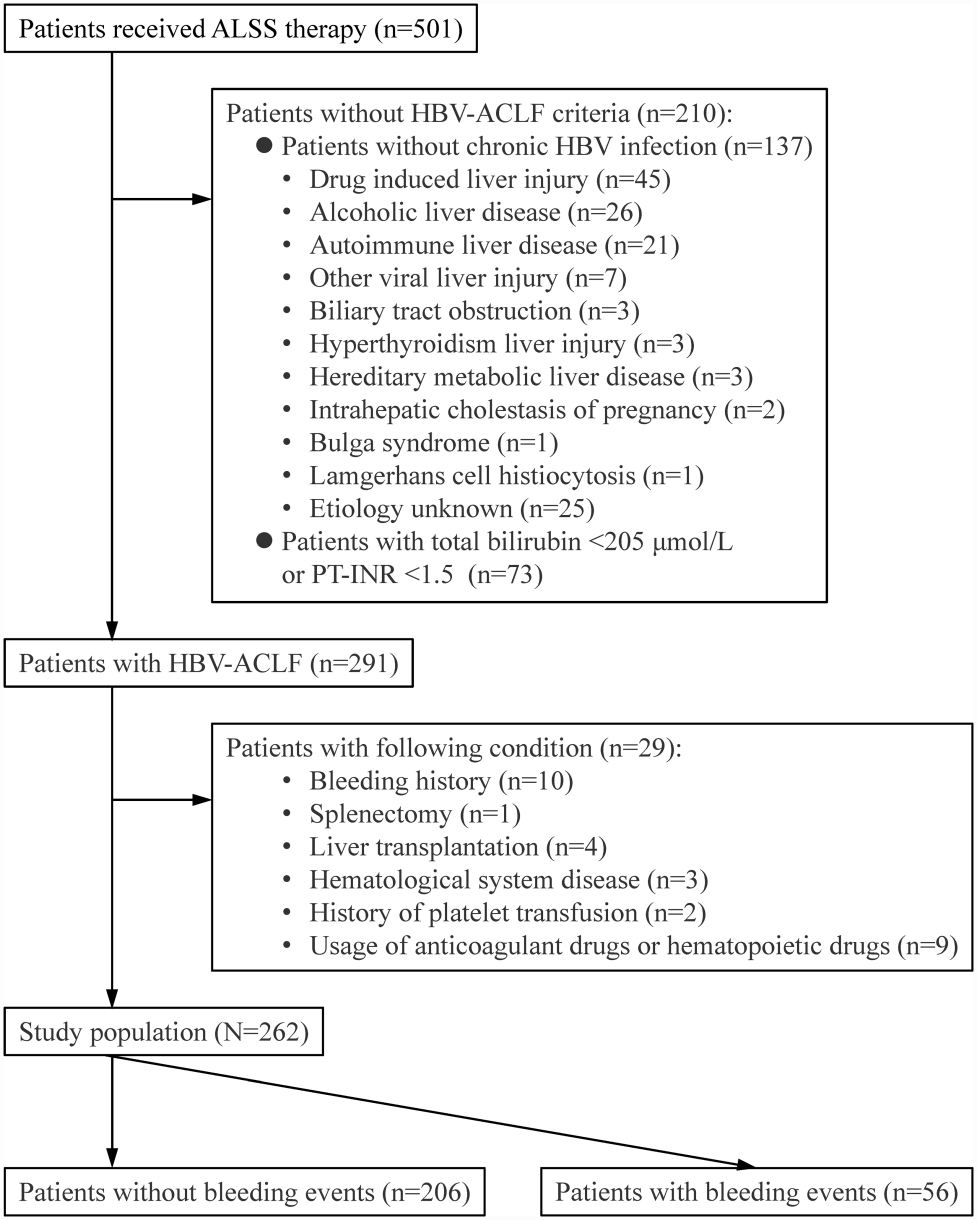
Patient selection and follow-up. ALSS, artificial liver support system; HBV, hepatitis B virus; COSSH, Chinese Group on the Study of Severe Hepatitis B; ACLF, acute-on-chronic liver failure; PT-INR, Prothrombin Time—International Normalized Ratio.

Patients with HBV-ACLF who underwent ALSS treatment were included in this study. HBV-ACLF was diagnosed based on the Chinese Group on the Study of Severe Hepatitis B (COSSH) ACLF criteria: regardless of the presence of cirrhosis, patients with chronic HBV infection, total bilirubin level of ≥ 12 mg/dL (205 μmol/L), and international normalized ratio of prothrombin time (PT-INR) of ≥1.5 could be diagnosed with ACLF (16). The exclusion criteria were as follows: (1) concurrent malignancies of the hematological system, such as hematologic tumors and bone marrow suppression, as well as the use of drugs with a known impact on bone marrow suppression or promotion of hematopoiesis; (2) congenital or acquired disorders of coagulation factors and fibrinogen causing PLT reduction; (3) disseminated intravascular coagulation; (4) the use of heparin-like drugs, anti-PLT drugs, and PLT transfusion within the first 7 days before ALSS treatment; (5) documented bleeding within the first 7 days before ALSS treatment; (6) history of splenectomy; (7) post-liver transplantation; and (10) pregnancy (Figure 1).

Patient clinical data, including demographic information (sex, age, etiology, etc.) and laboratory parameters (routine blood tests, liver and kidney function, coagulation parameters, HBV DNA, etc.), were collected before the initial ALSS treatment. Patients were followed up until the occurrence of bleeding events during hospitalization or until discharge in the absence of bleeding events. Detailed manifestations and types of bleeding events have been documented.

All patients received at least one session of ALSS treatment in addition to standard medical care, which included etiology-specific treatment, management of complications, nutritional support, and hepatoprotective therapy. ALSS treatment involves a double plasma molecular adsorption system (DPMAS) with sequential plasma exchange (PE) (17). PE treatment utilized 1,500 mL of plasma, and the anticoagulant used was 4% sodium citrate. ALSS treatment was scheduled once every 1–2 days.

### Definition

The PLT level before ALSS treatment was defined as the baseline PLT level and categorized into four grades: Grade 0 (PLT ≥ 100 × 10^9^/L), Grade 1 (PLT (50–99) × 10^9^/L), Grade 2 (PLT (20–49) × 10^9^/L), and Grade 3 (PLT < 20 × 10^9^/L) (18). The PLT level at the time of bleeding events or at the end of the final ALSS treatment in patients without bleeding events was defined as the final PLT level. The platelet reduction rate was calculated as the difference between the baseline and final PLT levels divided by the baseline PLT level.

Bleeding events were categorized into four grades based on the severity and clinical impact (19): grade 0, no bleeding events; grade 1, minor bleeding events such as skin petechiae, ecchymosis, epistaxis, gingival bleeding, oozing at the site of central venous catheterization, or bleeding at puncture points, requiring no intervention or only minor, local, or non-invasive treatment; grade 2, moderate bleeding events such as positive fecal occult blood, subcutaneous hematoma, or other bleeding events that led to prolonged hospitalization; and grade 3, severe or life-threatening bleeding events, including upper gastrointestinal bleeding and intracranial bleeding, which require urgent intervention or may result in death. For patients with multiple concurrent bleeding events, the most severe event was included in the analysis of patients with multiple concurrent bleeding events.

All patients underwent central venous catheterization. Patients with bleeding events who were associated with central venous catheterization and other invasive procedures were classified as “yes” for invasive procedures, whereas the others were classified as “no.” Similarly, patients who received PLT-increasing medications or PLT transfusions were classified as “yes” for PLT intervention, while the others were classified as “no.” Additionally, the patients were classified into three categories based on the number of ALSS treatment sessions received: 1 ~ 2 sessions, 3 ~ 5 sessions, and ≥6 sessions (20).

### Statistical analysis

Normally distributed continuous variables are presented as mean (standard deviation), and comparisons between groups were conducted using the *t*-test. Non-normally distributed continuous variables are presented as medians (*P*_25_–*P*_75_), and comparisons between groups were performed using the Wilcoxon rank-sum test. Categorical variables are presented as numbers (percentages), and comparisons between groups were performed using the *χ^2^* test. The Bonferroni correction was applied for multiple comparisons. Univariate and multivariate logistic regression analyses were performed to assess the relationship between baseline PLT levels, baseline PLT grades, PLT reduction rates, and bleeding events. Univariate and multivariate ordinal logistic regression analyses were performed to evaluate the relationship between baseline PLT grade and the grade of bleeding events. Univariate and multivariate linear regression analyses were conducted to assess the relationship between sessions of ALSS treatment and the PLT reduction rate.

Although this study was a retrospective analysis, the data consisted of routinely collected clinical variables, ensuring completeness with no missing values, and a complete case-analysis approach was applied. Data were analyzed using the Statistical Package for the Social Sciences (SPSS, version 25.0). Statistical significance was set at *p* < 0.05.

## Results

### Patient characteristics

A total of 262 patients with HBV-ACLF who received ALSS treatment were included in this study (Figure 1). Among these patients, 28 (10.7%) were women, and 208 (79.4%) had cirrhosis (Table 1). The Model for End-Stage Liver Disease (MELD) score was significantly higher in patients with bleeding events than in those without bleeding events (20.0 (16.3 ~ 23.5) vs. 16.4 (14.1 ~ 20.2), *p* < 0.001). Similarly, the PT-INR was markedly elevated in patients with bleeding events compared to those without bleeding events (2.3 (2.0 ~ 3.2) vs. 1.9 (1.7 ~ 2.4), *p* < 0.001).

**Table 1:** Patient characteristics.

Parameters	All patients (*n* = 262)	Patients with bleeding events (*n* = 56)	Patients without bleeding events (*n* = 206)	*P*
Gender				0.631
Female	28 (10.7%)	5 (8.9%)	23 (11.2%)	
Male	234 (89.3%)	51 (91.1%)	183 (88.8%)	
Age (years)	48.6 (11.1)	50.8 (10.7)	48.0 (11.1)	0.092
Etiology				0.083
HBV infection only	236 (90.1%)	47 (83.9%)	189 (91.7%)	
HBV infection plus other causes	26 (9.9%)	9 (16.1%)	17 (8.3%)	
Cirrhosis				0.198
No	54 (20.6%)	15 (26.8%)	39 (18.9%)	
Yes	208 (79.4%)	41 (73.2%)	167 (81.1%)	
HBV DNA (log10IU/ml)	4.6 (3.2 ~ 6.1)	4.2 (3.0 ~ 5.5)	4.7 (3.2 ~ 6.1)	0.280
MELD score	17.3 (14.5 ~ 21.1)	20.0 (16.3 ~ 23.5)	16.4 (14.1 ~ 20.2)	<0.001
Total bilirubin (μmol/L)	380.9 (311.4 ~ 454.7)	390.6 (321.3 ~ 482.6)	374.8 (309.0 ~ 446.1)	0.140
PT-INR	2.0 (1.7 ~ 2.5)	2.3 (2.0 ~ 3.2)	1.9 (1.7 ~ 2.4)	<0.001
Serum creatinine (μmol/L)	65.5 (56.0 ~ 83.0)	68.5 (57.5 ~ 83.0)	64.0 (56.0 ~ 82.3)	0.383
Baseline platelet (×10^9^/L)	82.0 (51.0 ~ 113.0)	59.0 (39.0 ~ 89.3)	88.5 (57.0 ~ 121.0)	<0.001
Baseline platelet grade^§^				<0.001
Grade 0	96 (36.6%)	8 (14.3%)	88 (42.7%)	
Grade 1	105 (40.1%)	24 (42.9%)	81 (39.3%)	
Grade 2	55 (21.0%)	22 (39.3%)	33 (16.0%)	
Grade 3	6 (2.3%)	2 (3.6%)	4 (1.9%)	
Final platelet (×10^9^/L)	55.5 (33.0 ~ 76.0)	37.5 (28.0 ~ 71.5)	57.0 (34.0 ~ 76.3)	0.017
Platelet reduction (×10^9^/L)	25.0 (10.0 ~ 47.3)	14.0 (5.0 ~ 24.8)	29.5 (11.8 ~ 53.3)	<0.001
Platelet reduction rate (%)	31.4 (17.1 ~ 48.9)	22.6 (13.7 ~ 40.7)	34.4 (20.4 ~ 51.3)	0.008
Sessions of ALSS treatment	4.0 (2.0 ~ 5.0)	1.0 (1.0 ~ 3.0)	4.0 (3.0 ~ 5.0)	<0.001
Stratified sessions of ALSS treatment				<0.001
1 ~ 2	86 (32.8%)	41 (73.2%)	45 (21.8%)	
3 ~ 5	123 (46.9%)	11 (19.6%)	112 (54.4%)	
≥6	53 (20.2%)	4 (7.1%)	49 (23.8%)	
Platelet intervention				>0.99
No	241 (92.0%)	52 (92.9%)	189 (91.7%)	
Yes	21 (8.0%)	4 (7.1%)	17 (8.3%)	
Invasive procedure				0.034
No	205 (78.2%)	38 (67.9%)	167 (81.1%)	
Yes	57 (21.8%)	18 (32.1%)	39 (18.9%)	

HBV, hepatitis B virus; MELD, Model for End-Stage Liver Disease; PT-INR, international normalized ratio of prothrombin time; ALSS, artificial liver support system.

Values are presented as mean (SD), median (P_25_–P_75_), or number of patients (percentage).

^§^ Platelet grades: Grade 0, platelet count ≥100 × 10^9^/L; Grade 1, platelet (50–99) × 10^9^/L; Grade 2, platelet (20–49) × 10^9^/L; Grade 3, platelet count <20 × 10^9^/L.

During hospitalization, patients received 4.0 (2.0 ~ 5.0) sessions of ALSS treatment; specifically, 86 (32.8%) patients underwent 1 ~ 2 sessions, 123 (46.9%) patients underwent 3 ~ 5 sessions, and 53 (20.2%) patients underwent 6 or more sessions (Table 1). During hospitalization, 56 patients (21.4%) experienced bleeding. During hospitalization, 56 patients (21.4%) experienced bleeding. Skin petechiae and ecchymosis were the most common manifestations, occurring in 21 patients (37.5% of all bleeding events). The majority of bleeding events were classified as grade 1 (37 patients, 66.1%), with grade 2 and grade 3 events observed in 11 (19.6%) and 8 (14.3%) patients, respectively.

The baseline PLT levels in patients with bleeding events were lower than those in patients without bleeding events (59.0 (39.0 ~ 89.3) × 10^9^/L vs. 88.5 (57.0 ~ 121.0) × 10^9^/L, *p* < 0.001). The PLT reduction rates were also lower in patients with bleeding events than in those without bleeding events (22.6% (13.7% ~ 40.7%) vs. 34.4% (20.4% ~ 51.3%), *p* = 0.008). Similarly, the final PLT levels in patients with bleeding events were lower than those in patients without bleeding events (37.5 (28.0 ~ 71.5) × 10^9^/L vs. 57.0 (34.0 ~ 76.3) × 10^9^/L, *p* = 0.017).

### Association between baseline platelet level, platelet reduction rate, and final platelet level and bleeding events

Baseline PLT levels were significantly associated with bleeding events in patients with ACLF who underwent ALSS treatment (crude odds ratio (OR) (95% confidence interval (CI)), 0.984 (0.976 ~ 0.993), *p* < 0.001) (Table 2, Model 1). After adjusting for sex, age, etiology, presence of cirrhosis, HBV DNA level, disease severity (MELD score or individual components: total bilirubin, PT-INR, serum creatinine), sessions of ALSS treatment (including session count or excluded), PLT reduction rate or final PLT level, PLT intervention, and invasive procedure, the baseline PLT level remained significantly associated with bleeding events (adjusted ORs (95% CIs): 0.980 ~ 0.987 (0.964 ~ 0.999), all *p* < 0.05) (Tables 2, 3, Models 2–4). In contrast, the PLT reduction rate (Table 2, Models 2–4) and final PLT levels (Table 3, Models 2–4) were not independently associated with bleeding events (all adjusted ORs (95% CIs) included 1, *p* > 0.05).

**Table 2:** Association between baseline platelet count and platelet reduction rate with bleeding events.

Logistic regression model	Baseline platelet (×10^9^/L)		Platelet reduction rate (%)	
	*OR* (95% CI)	*P*	*OR* (95% CI)	*P*
Model 1	0.984 (0.976 ~ 0.993)	<0.001	0.90 (0.57 ~ 1.42)	0.643
Model 2	0.986 (0.976 ~ 0.996)	0.006	0.94 (0.44 ~ 2.01)	0.872
Model 3	0.987 (0.977 ~ 0.997)	0.013	0.93 (0.41 ~ 2.11)	0.865
Model 4	0.984 (0.974 ~ 0.993)	0.001	1.21 (0.65 ~ 2.25)	0.557

OR, odds ratio; 95% CI, 95% confidence interval; MELD, Model for End-Stage Liver Disease; PT-INR, international normalized ratio of prothrombin time; ALSS, artificial liver support system.

Model 1 was not adjusted for any variable.

Model 2 was adjusted for sex, age, etiology, presence of cirrhosis, HBV DNA, MELD score, baseline platelet count, platelet reduction rate, platelet intervention, invasive procedures, and sessions of ALSS treatment.

Model 3 was adjusted for sex, age, etiology, presence of cirrhosis, HBV DNA, total bilirubin, PT-INR, serum creatinine level, baseline platelet count, platelet reduction rate, platelet intervention, presence of invasive procedures, and sessions of ALSS treatment.

Model 4 was adjusted for sex, age, etiology, presence of cirrhosis, HBV DNA, total bilirubin, PT-INR, serum creatinine level, baseline platelet count, platelet reduction rate, platelet intervention, and invasive procedures.

**Table 3:** Association of baseline and final platelet counts with bleeding events.

Logistic regression model	Baseline platelet (×10^9^/L)		Final platelet (×10^9^/L)	
	OR (95% CI)	*P*	OR (95% CI)	*P*
Model 1	0.984 (0.976 ~ 0.993)	<0.001	0.99 (0.98 ~ 1.00)	0.041
Model 2	0.981 (0.964 ~ 0.997)	0.021	1.01 (0.99 ~ 1.03)	0.439
Model 3	0.982 (0.965 ~ 0.999)	0.033	1.01 (0.99 ~ 1.03)	0.451
Model 4	0.980 (0.968 ~ 0.993)	0.002	1.01 (0.99 ~ 1.02)	0.282

OR, odds ratio; 95% CI, 95% confidence interval; MELD, Model for End-Stage Liver Disease; PT-INR, international normalized ratio of prothrombin time; ALSS, artificial liver support system.

Model 1 was adjusted for none.

Model 2 was adjusted for sex, age, etiology, presence of cirrhosis, HBV DNA level, MELD score, baseline platelet count, final platelet count, platelet intervention, invasive procedures, and sessions of ALSS treatment.

Model 3 was adjusted for sex, age, etiology, presence of cirrhosis, HBV DNA level, total bilirubin level, PT-INR, serum creatinine level, baseline platelet count, final platelet count, platelet intervention, invasive procedures, and sessions of ALSS treatment.

Model 4 was adjusted for sex, age, etiology, presence of cirrhosis, HBV DNA level, total bilirubin level, PT-INR, serum creatinine level, baseline platelet count, final platelet count, platelet intervention, and invasive procedures.

### Association between baseline platelet grades and bleeding events

Compared to patients with baseline PLT grade 0, those with baseline PLT grade 1 (crude OR (95% CI): 3.26 (1.39 ~ 7.67), *p* = 0.007) and grade 2 (crude OR (95% CI):7.33 (2.97 ~ 18.09)) had a higher risk of bleeding (Table 4, Model 1). After adjusting for sex, age, etiology, presence of cirrhosis, HBV DNA level, disease severity (MELD score or individual components: total bilirubin, PT-INR, serum creatinine), sessions of ALSS treatment (included as session count or excluded), PLT reduction rate, PLT intervention, and invasive procedure, higher risk of bleeding remained significant for patients with baseline PLT grade 1 (adjusted ORs (95% CIs): 3.21 ~ 3.76 (1.20 ~ 9.50), *p* < 0.05) and grade 2 (adjusted ORs (95% CIs): 7.20 ~ 8.40 (2.28 ~ 24.09), *p* ≤ 0.001), compared to those with grade 0 (Table 4, Models 2–3). Similarly, high risk of bleeding was also observed in patients with baseline PLT grades 2 and 3 (adjusted OR (95% CI): 8.43 (2.96 ~ 23.99), *p* < 0.001) (Table 4, Model 4).

**Table 4:** Association between baseline platelet grades and bleeding events.

Logistic regression model	Platelet Grade 0	Platelet Grade 1		Platelet Grade 2		Platelet Grade 3	
		OR (95% CI)	*P*	OR (95% CI)	*P*	OR (95% CI)	*P*
Model 1	1 (Ref)	3.26 (1.39 ~ 7.67)	0.007	7.33 (2.97 ~ 18.09)	<0.001	5.50 (0.87 ~ 34.81)	0.070
Model 2	1 (Ref)	3.21 (1.20 ~ 8.59)	0.020	7.20 (2.28 ~ 21.43)	0.001	5.59 (0.54 ~ 58.29)	0.150
Model 3	1 (Ref)	3.76 (1.49 ~ 9.49)	0.005	8.40 (2.93 ~ 24.09)	<0.001	8.80 (0.95 ~ 81.41)	0.055
Model 4	1 (Ref)	3.76 (1.49 ~ 9.50)	0.005	8.43 (2.96 ~ 23.99)^&^	<0.001	–	

OR, odds ratio; 95% CI, 95% confidence interval; MELD, Model for End-Stage Liver Disease; PT-INR, international normalized ratio of prothrombin time; ALSS, artificial liver support system.

^&^: combined baseline platelet grades 2 and 3.

Model 1 was adjusted for none.

Model 2 was adjusted for sex, age, etiology, presence of cirrhosis, HBV DNA, MELD score, baseline platelet grade (grade 1 vs. 0, 2 vs. 0, and 3 vs. 0), platelet reduction rate, platelet intervention, invasive procedures, and sessions of ALSS treatment.

Model 3 was adjusted for sex, age, etiology, presence of cirrhosis, HBV DNA, total bilirubin, PT-INR, serum creatinine, baseline platelet grade (grade 1 vs. 0, 2 vs. 0, and 3 vs. 0), platelet reduction rate, platelet intervention, invasive procedure, and sessions of ALSS treatment.

Model 4 was adjusted for sex, age, etiology, presence of cirrhosis, HBV DNA, total bilirubin, PT-INR, serum creatinine, baseline platelet grade (grade 1 vs. 0, 2, and 3 vs. 0), platelet reduction rate, platelet intervention, and invasive procedures.

### Association between baseline platelet grades and bleeding event grades

Among the 56 patients (21.4%) with bleeding events, 37 (14.1%), 11 (4.2%), and 8 (3.1%) had grade 1, 2, and 3 bleeding events, respectively. A significantly higher incidence of bleeding events was observed in patients with baseline PLT grades 1, 2, and 3 compared to those with grade 0 PLTs (22.9% vs. 8.3, 40.0% vs. 8.3, and 33.3% vs. 8.3%, respectively, all having a *p* value of <0.05). Furthermore, patients with baseline PLT grades 2 and 3 had a higher incidence of bleeding events than those with grade 0 PLTs (39.3% vs. 8.3%, adjusted *p* < 0.05).

Compared to baseline PLT grade 0, baseline PLT grade 2 was significantly associated with a higher bleeding event grade (crude OR (95% CI): 3.06 (1.61 ~ 5.84), *p* = 0.001) (Table 5, Model 1). After adjusting for sex, age, etiology, the presence of cirrhosis, HBV DNA level, disease severity (MELD score or individual components: total bilirubin, PT-INR, serum creatinine), the number of ALSS treatment sessions (included as session count or excluded), PLT reduction rate, PLT intervention, and invasive procedure, the results indicated that baseline PLT grade 2 (adjusted ORs (95% CIs): 4.23 ~ 4.91 (1.47 ~ 14.04), all *p* < 0.01) and the combined category of baseline PLT grades 2 and 3 (adjusted OR (95% CI): 6.83 (2.49 ~ 18.67), *p* < 0.001) remained significantly associated with bleeding event grade compared to grade 0 (Table 5, Model 2–4).

**Table 5:** Association between baseline platelet grades and bleeding event grades.

Ordinal regression model	Platelet Grade 0	Platelet Grade 1		Platelet Grade 2		Platelet Grade 3	
		OR (95% CI)	*P*	OR (95% CI)	*P*	OR (95% CI)	*P*
Model 1	1 (Ref)	1.16 (0.64 ~ 2.10)	0.631	3.06 (1.61 ~ 5.84)	0.001	2.11 (0.40 ~ 11.02)	0.377
Model 2	1 (Ref)	2.54 (1.00 ~ 6.46)	0.051	4.23 (1.47 ~ 12.21)	0.008	3.43 (0.38 ~ 30.60)	0.271
Model 3	1 (Ref)	2.60 (1.03 ~ 6.56)	0.044	4.91 (1.72 ~ 14.04)	0.003	5.65 (0.64 ~ 49.95)	0.120
Model 4	1 (Ref)	3.48 (1.42 ~ 8.52)	0.006	6.83 (2.49 ~ 18.67)^&^	<0.001	–	

OR, odds ratio; 95% CI, 95% confidence interval; PT-INR, international normalized ratio of prothrombin time; ALSS, artificial liver support system.

^&^: combined baseline platelet grades 2 and 3.

Model 1 was adjusted for none.

Model 2 was adjusted for sex, age, etiology, presence of cirrhosis, HBV DNA, MELD score, baseline platelet grade (grade 1 vs. 0, 2 vs. 0, 3 vs. 0), platelet reduction rate, platelet intervention, invasive procedures, and sessions of ALSS treatment.

Model 3 was adjusted for sex, age, etiology, presence of cirrhosis, HBV DNA, total bilirubin, PT-INR, serum creatinine, baseline platelet grade (grade 1 vs. 0, 2 vs. 0, and 3 vs. 0), platelet reduction rate, platelet intervention, invasive procedure, and sessions of ALSS treatment.

Model 4 was adjusted for sex, age, etiology, presence of cirrhosis, HBV DNA, total bilirubin, PT-INR, serum creatinine, baseline platelet grade (grade 1 vs. 0, 2, and 3 vs. 0), platelet reduction rate, platelet intervention, and invasive procedures.

### Association between ALSS treatment sessions and platelet reduction rates

Sessions of ALSS treatment were not significantly associated with the PLT reduction rate (crude *β* (95% CI): −0.011 (−0.041 ~ 0.020), *p* = 0.490). After adjusting for sex, age, etiology, presence of cirrhosis, HBV DNA level, total bilirubin, PT-INR, serum creatinine, baseline PLT level, PLT intervention, and invasive procedure, there was no significant association between sessions of ALSS treatment and the PLT reduction rate (adjusted β (95% CI): −0.021 (−0.053–0.011), *p* > 0.05).

## Discussion

Patients with chronic liver disease may experience varying levels of PLT reduction, with a more pronounced reduction observed in those with liver failure (8–13). PLT reduction is also associated with disease progression, including the worsening of liver failure, the onset of multiorgan dysfunction, and adverse clinical outcomes (21, 22). Although ALSS treatment is an important therapeutic method for liver failure, it may also contribute to further reductions in platelet levels (23, 24). However, the relationship between PLT levels, their reduction, and bleeding events in patients with ACLF who underwent ALSS treatment remains unclear. Our findings indicate that the baseline PLT level, but not the platelet reduction rate, was an independent risk factor for bleeding events during hospitalization in patients with ACLF receiving ALSS treatment. Additionally, patients with baseline PLT levels below 50 × 10^9^/L had a significantly higher risk of bleeding than those with baseline PLT levels of 100 × 10^9^/L. These findings are consistent with those reported in several previous studies, with a primary distinction in the study populations (25–27). A prospective study involving 1770 cases of acute liver failure found that reduced platelet counts were associated with bleeding complications during hospitalization (25). The majority of patients experienced spontaneous bleeding in the upper gastrointestinal tract, and approximately 5% of them died as a result (25). Another study involving 589 patients with hematologic malignancies found that patients with PLT ≤ 50 × 10^9^/L had a higher risk of WHO bleeding grades 2 ~ 4 compared to those with PLT > 50 × 10^9^/L (PLT: (41 ~ 50) × 10^9^/L, hazard ratio (HR) (95% CI): 2.57(0.56 ~ 11.77); PLT: (31 ~ 40) × 10^9^/L, HR(95% CI): 2.74(0.74 ~ 10.16); PLT: (21 ~ 30) × 10^9^/L, HR(95% CI): 2.25(0.67 ~ 7.61); PLT: ≤20 × 10^9^/L, HR(95% CI): 9.77(3.35 ~ 28.54)) (26). A similar association was found in cirrhotic patients with Child-Pugh B or C and baseline PLT grade 3, who exhibited an increased risk of post-polypectomy bleeding after colonoscopy (27). In our study of patients with the COSSH ACLF score, the majority of bleeding events were grade 1 (14.1%), with only a small proportion classified as grade 2 (4.2%) or grade 3 (3.1%). Nevertheless, baseline PLT levels had a significant influence on the severity of bleeding. Therefore, clinical attention should be paid to the management of patients undergoing ALSS treatment with lower baseline PLT levels (i.e., higher thrombocytopenia grades).

The dissociation between the platelet reduction rate and bleeding events underscores the need for mechanistic investigations beyond simple PLT counting. To interpret these findings, the concept of “rebalanced hemostasis” in liver failure provides an essential framework. In chronic liver disease, PLT reduction may be partially compensated for by elevated levels of von Willebrand factor (vWF), which enhances PLT adhesion and aggregation at sites of vascular injury, thereby promoting coagulation (28). However, the hemostatic system in ACLF is complex and dynamic. This state of “rebalanced hemostasis” implies that traditional static coagulation tests, such as PLT level and PT-INR, are unreliable predictors of bleeding risk, particularly in patients undergoing ALSS treatment. The underlying mechanisms are driven by specific pathophysiological mechanisms. Systemic inflammation, a hallmark of ACLF, induces endothelial activation and the release of large vWF multimers, whereas impaired hepatic synthesis leads to reduced levels of ADAMTS-13, the protease responsible for its cleavage. This “high vWF/low ADAMTS-13” axis enhances PLT adhesiveness, potentially offsetting the numerical PLT deficit (29). This mechanism offers a coherent explanation for our results: baseline PLT levels may reflect an individual’s established, albeit fragile, hemostatic balance, thus retaining their prognostic value. In contrast, acute procedural decline during ALSS treatment may be functionally mitigated by this compensatory pathway, accounting for the lack of an independent association with bleeding events.

This pathophysiological framework also elucidates the dual role of ALSS treatment. The extracorporeal circulation circuit, filter, and perfusion equipment used in blood purification may contribute to PLT reduction through mechanisms such as blood coagulation, mechanical destruction of blood cells, and biological incompatibility reactions, thereby influencing disease severity and patient outcomes (23). However, ALSS treatment could also exert anti-inflammatory effects by removing inflammatory mediators, which may help mitigate thrombocytopenia (30, 31). In this study, we found no significant association between sessions of ALSS treatment and the PLT reduction rates. We speculate that these opposing effects reach a net equilibrium, accounting for our observations.

Collectively, these insights have refined the clinical implications. First, the identification of high-risk patients should incorporate the baseline thrombocytopenia severity. Second, the “rebalanced” hemostatic state implies that reliance on static tests for procedural decision-making is inherently limited. The safe threshold of PLT levels varies according to multiple factors, such as the type of procedure or surgery, operator expertise, inherent bleeding risk of the intervention, and concomitant coagulation status, rather than being defined by a single universal threshold (32). Prophylactic PLT transfusion should be routinely performed only before specific, high-risk invasive procedures (32, 33). In our study, neither the PLT reduction rate nor the final PLT level was an independent risk factor for bleeding events, and the PLT intervention showed no significant association with bleeding events. Crucially, our finding that both the PLT reduction rate and final PLT level lacked independent predictive value, coupled with the absence of benefit from platelet intervention, strongly argues against the prophylactic use of PLT-inducing drugs or transfusions based solely on PLT levels during ALSS treatment (34). Recent studies have shown that PLT reduction is proportional to the severity of the systemic inflammatory response and complications in patients with liver failure (25, 28). Bleeding events are usually associated with portal hypertension, endothelial dysfunction (vasoconstriction), renal failure, recurrent bacterial infections, endogenous heparin-like substance production, and disseminated intravascular coagulation, and may serve as indicators of severe systemic inflammation (25, 28). Therefore, clinical attention should be focused on managing the underlying risk factors in patients with ACLF undergoing ALSS treatment to reduce the risk of bleeding during hospitalization.

This study had several limitations. First, it was a single-center retrospective study with a relatively small sample size, which might limit the generalizability of the findings and potentially introduce a selection bias. Specifically, owing to the limited sample size, the risk of overfitting may increase. A limitation associated with the retrospective design was that minor bleeding events (e.g., skin petechiae, ecchymosis, epistaxis, and gingival bleeding) might not have been consistently or completely documented in the clinical records, which could have led to an underestimation of the overall bleeding incidence. Second, the study population was limited to patients with COSSH ACLF who underwent ALSS treatment with citrate anticoagulation. Therefore, the results may not be generalizable to patients without HBV infection, those undergoing different anticoagulant strategies, or those without ALSS. It should be noted that the COSSH ACLF criteria serve as an additional diagnostic criterion for ACLF alongside the EASL-ACLF criteria and differ from them in their diagnostic components and patient population definitions. Furthermore, since citrate anticoagulation was uniformly administered to all patients, it could not serve as a confounding variable when assessing the differential risk of bleeding events across different PLT levels. Third, in the multivariate adjustment models, as many clinically relevant variables as possible were included. Nevertheless, the presence of unmeasured confounders was inevitable and may have influenced the study findings. Specifically, PLT function, comprehensive coagulation profiles (such as thromboelastography, vWF, and ADAMTS-13 activity), comorbid conditions, severity of cirrhosis, and indicators of portal hypertension were not evaluated due to their unavailability in this retrospective study. These markers are crucial for evaluating complex and rebalanced coagulation states in patients with ACLF. Therefore, although the baseline PLT level was identified as an independent risk factor, the lack of functional data limited our ability to elucidate the underlying pathophysiology. It remains unclear whether bleeding risk is primarily driven by the PLT quantity, function, or a broader, rebalanced hemostatic milieu. Fourth, although Cox regression is the preferred method for survival or time-to-event analysis, logistic regression was employed in this study as an alternative approach to assess in-hospital adverse events, which may have introduced potential bias.

In summary, this study clarified the relationship between PLT dynamics and bleeding risk in a specific high-risk cohort of patients with ACLF who underwent ALSS treatment. Our key finding was that the baseline PLT level, but not platelet reduction rate, was an independent risk factor for bleeding events during hospitalization in patients with ACLF receiving ALSS treatment. This distinction has direct clinical implications, indicating that bleeding risk stratification should prioritize patients with significant thrombocytopenia. Importantly, the lack of an association between procedural PLT reduction and bleeding events argues against routine prophylactic PLT transfusion based solely on intra-treatment PLT reduction, thereby supporting more rational and evidence-based transfusion strategies. Beyond its immediate clinical utility, this finding refines our pathophysiological understanding of hemostasis in ACLF. The unique prognostic value of the baseline PLT level—compared to its dynamic changes—suggests that it may serve as a surrogate marker for the patient’s underlying, potentially compromised, compensatory capacity within the “rebalanced” hemostatic state. This highlights that bleeding risk is governed by a complex systemic equilibrium rather than the PLT level alone. Future large-scale, multicenter prospective studies are needed to validate these findings and to advance the observed associations with mechanistic insights. Such investigations should incorporate functional coagulation assessments, such as viscoelastic testing (VET), along with specific molecular markers (e.g., vWF and ADAMTS-13) to delineate the relative contributions of PLT quantity, PLT function, and systemic coagulopathy. Integrated approaches of this type are essential for developing a more comprehensive and pathophysiologically grounded risk-stratification framework, ultimately enabling the personalized management of this vulnerable population.

## Data Availability

The original contributions presented in the study are included in the article/supplementary material, further inquiries can be directed to the corresponding authors.

## Footnotes

1
https://www.chictr.org.cn/showproj.html?proj=208697
